# Barriers and facilitators of direct access model to physiotherapy: intervention design and implementation strategies using the consolidated framework for implementation research

**DOI:** 10.1186/s13690-026-01882-7

**Published:** 2026-03-12

**Authors:** Carrie Ho-Kwan Yam, Eng-Kiong Yeoh, Ethan Ming-Yin Ip, Tsz-Yu Chow, Eliza Lai-Yi Wong, Chi-Tim Hung

**Affiliations:** Centre for Health Systems and Policy Research, JC School of Public Health and Primary Care, Faculty of Medicine, The Chinese University of Hong Kong, Hong Kong SAR, China

**Keywords:** Physical therapy, Primary care, Implementation science, Policy design, Rehabilitation

## Abstract

**Background:**

Although the benefits of direct access to physiotherapy are well-documented, challenges in the policy design remain in the context of patient safety and organizational readiness. This study aims to identify barriers and facilitators for implementing the direct access to physiotherapy policy, with a goal of informing the development of policy design options and implementation strategies acceptable to key stakeholders.

**Methods:**

This qualitative study is a component of a sequential mixed-methods design. Key informant interviews and focus groups were conducted with stakeholders including policymakers, tertiary physiotherapy education academics, employers, physiotherapists, doctors and patients. Data were analyzed using the Consolidated Framework for Implementation Research (CFIR).

**Results:**

Physiotherapists and patients highlighted evidence supporting improved accessibility, service efficiency, and better health outcomes associated with direct access. Patients valued improved access and greater choice, while also emphasising the importance of safety and quality assurance. Physiotherapists expressed confidence to perform physical diagnosis, make appropriate referrals, and maintain competence through continuous professional development, and in their ability gained through undergraduate and postgraduate training to identify red flags. Doctors expressed mixed views, with some emphasizing the need for a medical diagnosis prior to physiotherapy access to ensure safety. Key barriers included limited understanding of the government’s proposal, insufficient stakeholder engagement, and concerns regarding patient safety. Facilitators included improving public knowledge, ensuring physiotherapist competency, and clarifying the roles and scope of physiotherapy.

**Conclusions:**

Successful implementation of a direct access model requires appropriate policy design, clear protocols, recognition of professional judgement and autonomy, and robust safety measures. Continuous evaluation, public education, and stakeholder engagement are essential to support policy effectiveness and build public trust. These findings provide a foundation for developing an appropriate and acceptable policy design and context-specific implementation strategies, which will be further refined through expert consensus in a subsequent Delphi study.

**Supplementary Information:**

The online version contains supplementary material available at 10.1186/s13690-026-01882-7.

**Table:** 

Text box 1. Contributions to the literature	
• Expands global understanding of implementing direct access models to physiotherapy in traditionally physician-led health systems, where evidence remains limited.	
• Demonstrates the utility of implementation science frameworks (CFIR) for designing context-specific policies and corresponding implementation strategies in allied health care.	
• Highlights the importance of balancing professional autonomy with patient safety in policy development for allied health services.	
• Provides actionable insights for policymakers seeking to adapt international best practices to local contexts without compromising system integrity.	

## Background

In many healthcare systems across Asia, including Hong Kong, patients are required to obtain a referral from a registered medical practitioner before accessing physiotherapy services. This “gatekeeping” model contrasts with the practice in other countries such as the United Kingdom, United States, and Australia, where direct access to physiotherapy—also known as patient self-referral—is permitted under defined conditions, such as through regulatory mechanisms and professional guidelines or insurance requirements [1–3]. Evidence suggests that direct access can reduce waiting times, improve health outcomes, and enhance system efficiency, particularly for musculoskeletal conditions [4–8]. However, concerns regarding patient safety, professional competency, and the readiness of healthcare systems need to be considered when implementing direct access.

The Hong Kong Special Administrative Region has proposed legislative amendments to allow direct access to physiotherapy in primary care settings [9, 10]. This policy shift aligns with broader efforts to strengthen primary healthcare and optimize the role of allied health professionals. Nevertheless, the proposed model has generated debate among key stakeholders, particularly regarding the scope of practice, regulatory safeguards, and implementation feasibility.

Despite the growing international evidence supporting direct access to physiotherapy, there remains a significant gap in understanding how the model can be effectively adapted and implemented in health systems with traditional physician-led referral systems, such as Hong Kong. Most existing studies have focused on clinical outcomes or cost-effectiveness in Western contexts [5, 6], with limited studies on the policy, organizational, and professional dynamics that influence implementation in the context of other healthcare systems, particularly in jurisdictions with traditional physician-led referral systems. Furthermore, few studies have systematically applied implementation science frameworks—such as the Consolidated Framework for Implementation Research (CFIR)—to examine stakeholder perspectives and contextual determinants that need to be considered in the policy design and implementation [11]. This study addresses these gaps by providing an in-depth qualitative analysis of the policy design and barriers and facilitators in the implementation of direct access to physiotherapy in Hong Kong, offering context-specific insights that can inform acceptable policy design and implementation strategies needed in contested settings.

This study is a part of the systemic study for “the policy intervention design and development of the implementation strategies for direct access to physiotherapists in primary care: a sequential mixed-method study using implementation mapping and a Delphi survey” [12]. This paper presents findings from the qualitative component of the study, which aimed to explore the perspectives of the key stakeholders - including policymakers, tertiary education institutions, healthcare professional bodies and professionals, and patients - on the barriers and facilitators in the implementation of direct access to physiotherapy, analyzed using the CFIR [11]. The findings can provide critical insights into the contextual factors that must be addressed to ensure the safe, effective, and acceptable design and implementation of direct access to physiotherapy services in Hong Kong.

### Conceptual framework

This study employed the CFIR to systematically analyze the barriers and facilitators influencing the implementation of direct access to physiotherapy [11]. CFIR provides a theoretical construct of five key domains -intervention characteristics, outer setting, inner setting, characteristics of individuals, and process of implementation - which offer a structured lens to examine complex contexts that are critical for implementation outcomes. The intervention characteristics of the policy design will affect the implementation factors in the other domains and the subsequent implementation strategies needed. In this study, CFIR enabled the mapping of stakeholder perspectives from key informant interviews and focus group discussions to the specific constructs, revealing nuanced insights in the implementation landscape. By applying CFIR, the study was able to subsequently map corresponding targeted implementation strategies to address the barriers aligned with the contextual determinants, and also informing a more appropriate and acceptable policy design.

## Methods

### Data collection method

To explore stakeholder perspectives on the policy design and implementation of direct access to physiotherapy, we conducted qualitative data collection through (i) key informant interviews, and (ii) focus group discussions. These methods aimed to capture in-depth insights into the perceived needs, barriers, facilitators, and contextual considerations for the proposed direct access model.

#### Key informant interviews

Key informant interviews were conducted involving a diverse range of key stakeholders who have extensive experience and leadership strategic role, including government policy decision-makers (*n* = 2), representatives from the regulatory body (Physiotherapists Board) (4), the medical specialist training authority (Academy of Medicine) (3), professional associations (physiotherapy and medical) (3), provider organizations (3), insurance agencies (3), tertiary education institutions offering physiotherapy programs (4), and patient advocacy groups (5). Participants were selected using a maximum variation sampling strategy to ensure heterogeneity in perspectives and experiences [13], and recruited by email. Inclusion criteria included individuals with relevant expertise or decision-making roles, the representatives of the patient advocacy groups who had direct experience of physiotherapy services.

Each interview lasted approximately 60 min, and was conducted at the interviewee’s office. Before the interview, the principal investigator or a co-investigator introduced themselves and the objectives of the study, and a researcher documented key points throughout the interview. Interview guides were developed for this study from the literature review and tailored to each stakeholder group and pilot-tested prior to use (Supplementary file 1). Topics included views on the necessity, feasibility and conditions for direct access, criteria for eligibility, safety and quality concerns, and anticipated implementation challenges. Interviews were audio-recorded with the subjects’ consent and transcribed verbatim for analysis. The interviews were conducted until data saturation was reached, meaning no new information or themes emerged.

#### Focus group discussions

Focus group discussions were held with frontline practicing staff and patients with prior experience of receiving physiotherapy services to complement the key informant interviews and facilitate dialogue among stakeholders with shared or contrasting experiences. Participants were grouped into three categories: (1) adopters (patients who had received physiotherapy services, grouped by post-discharge patients, community dwelling patients, and a mix of post-discharged and community patients), (2) providers (physiotherapists from public sector, private sector, and a mix of both public and private sectors), and (3) referrers (doctors who refer patients to physiotherapy, grouped by inpatient setting, outpatient and community setting, and a mix of both settings). Quota sampling ensured representation across sectors, specialties, and demographic backgrounds. Focus group participants were different from key informant interviews, allowing inclusion of a broader range of stakeholders and enhancing the diversity of perspectives. Doctors and physiotherapists were recruited via email invitations sent through their respective professional associations, while patients were recruited through patient groups and the university’s mass mail system. The recruitment email included a brief introduction of study, and participants were asked to complete a short questionnaire capturing demographic and background information relevant to focus group allocation (e.g., for patients: service setting and clinical conditions for seeing physiotherapists; for doctors and physiotherapists: type of practice and work setting).

Each focus group included 4–6 participants and lasted 90–120 min, and was conducted either at researchers’ office or by zoom. A discussion guide was developed from the literature examining barriers and facilitators [14, 15] and from the findings of the key informant interview findings, aligned with the CFIR (Supplementary file 2). Topics included service demand, professional readiness, training needs, interprofessional collaboration, and patient safety. Sessions were conducted by trained co-investigator to introduce themselves and the objectives of the study, and a researcher documented key points throughout the discussion. Focus group discussions were conducted until data saturation was reached. All discussions were audio-recorded and transcribed verbatim.

### Data analysis

Initial analysis of the key informant interview was conducted to identify emerging themes which informed the development of the focus group discussion guides. Following completion of all interviews and focus groups, the full dataset was analyzed thematically using a coding framework informed by CFIR constructs. Dedoose software was used for coding and managing the analysis. To enhance the reliability, a group analytical approach was applied. Two research staff independently created codes for 20% of the transcripts. They then clustered these codes into potential themes and sub-themes. Next, they met and agreed on the potential codes and themes. As they continued reading the transcripts separately, they repeatedly compared and contrasted their coding schemas to identify new themes and codes. This iterative process led to a consensus-based interpretation of the data, ensuring consistency and a comprehensive understanding of the ideas presented during interviews and focus group discussions. Recurrent themes that emerge among interviewees across and within different focus groups were noted, merged, and refined under a master theme as the analysis progressed. Interpretations of these themes were illustrated with extracts from the transcripts. We adhered to the Consolidated Criteria for Reporting Qualitative Research (COREQ), a 32-item checklist, for reporting the results of the interviews and focus groups for reporting [16].

### Ethical issues/statement

This study was approved by the Survey and Behavioral Research Ethics Committee of The Chinese University of Hong Kong (SBRE-22-0054). After the study was explained to all participants, those taking part in face-to-face interviews or discussions provided written informed consent. For those participating via Zoom, verbal informed consent was obtained in accordance with approved ethical procedures.

## Results

### Participants’ profile

Between November 2023 and May 2024, we conducted key informant interviews with 27 stakeholders, including government decision-makers (*n* = 2), representatives from Physiotherapist Board (4), the Academy of Medicine (3), professional associations in physiotherapy and medicine (3), provider organizations spanning public, private and non-governmental sectors (3), insurance agencies (3), tertiary institutions offering physiotherapy programs (4), and key patient advocacy groups (5). All but two of the key informant interviews were completed prior to the focus group discussions. The exceptions were the two interviews with the insurance agency representatives in late May 2024 due to scheduling constraints.

From April to September 2024, seven focus groups with a total of 27 participants, comprising patients with prior physiotherapy experience, physiotherapists from public, private and non-governmental sectors, and doctors (Table 1). Additionally, individual interviews were conducted with 10 doctors and three patients who were unable to participate in the focus groups.

**Table 1:** Profile of focus groups participants

Stakeholders	Groupings	Participant Characteristics (no. of participants shown in brackets)
Patients	15 participants in 3 focus group discussions and 3 individual interviews	• Age: 18–40 (5); ≥41 (10) • Gender: Female (9); Male (6) • Type of patients: Post-discharge patients from hospitals (6); Patients in the community (9) • Consultation reasons: Musculoskeletal condition (9); Post-operative rehabilitation (6)
Physiotherapists	13 participants in 3 focus group discussions	• Working experience: <10 years (5); ≥10 years (8) • Gender: Female (5); Male (8) • Work setting: Public sector and Non-Governmental Organizations (7); Private sector (4); Both the public and private sector (2)
Medical doctors	12 participants in 1 focus group discussion and 10 individual interviews	• Working experience: <10 years (2); ≥10 years (10) • Gender: Female (2); Male (10) • Specialist (11) in Cardiovascular (1), Orthopaedics (3), Neurology (1), Geriatrics, Family Medicine (3), Pain (1), Rehabilitation (1), Emergency (1) • Work setting: Public sector (8); Private Sector (4) • Services setting: Both inpatient & outpatient setting (6); Outpatient & community setting (6) • Type of practice (for those working in private sector): Group practices (2) and solo practitioners (2)

Among the 15 patients recruited, 10 were aged 41 years or older. The primary reasons for seeking physiotherapy services included musculoskeletal conditions such as chronic low back pain, neck pain and osteoarthritis of knee, as well as post-operative rehabilitation following joint replacement, spine, or other major orthopaedic surgical procedures.

Of the 13 physiotherapists interviewed, eight had at least 10 years of professional experience. Similarly, among the 12 doctors, 10 had more than a decade of clinical experience, and 11 held specialist qualifications across various fields including cardiology, orthopaedics, neurology, geriatrics, family medicine, pain management, rehabilitation, and emergency medicine.

### Intervention design, barriers and facilitators in implementing a direct access model for physiotherapy

This study identified key facilitators and barriers in a model of a direct access to physiotherapy, organized under three overarching themes: (1) perceived need for the model, (2) policy design options, and (3) implementation barriers and facilitators (Table 2). The findings are mapped to relevant domains of the CFIR, providing a structured understanding of the contextual and systemic factors influencing the model’s acceptability and feasibility. Sample quotes identified by citation number are shown in Table 3.

**Table 2:** A visualization of research theme and CFIR domains that emerged as facilitators and barriers for PT direct access model

Themes categorised into CFIR domains	Facilitators/ Barriers	Elaboration
(A) Perceived need for PT direct access		
Intervention characteristics *(Intervention source*,* Evidence strength & quality*,* Relative advantage*,* Adaptability)*	Facilitators	1) Few or no adverse events reported in countries operating the direct access model 2) Evidence of better health outcomes, improved access, greater efficiency, and cost savings 3) Hong Kong-trained physiotherapists meeting international standards
	Barriers	4) Difficult transitioning from a long-established doctor-led referral practice to a direct access model to allied health services
Outer Setting *(Patient needs & resources*,* External Policy & Incentives*,* Peer Pressure)*	Facilitators	1) Healthcare system challenges in Hong Kong (e.g., ageing population, chronic condition burden, early treatment and shorter waiting times in public sector) 2) Patient autonomy and choice 3) Government support and legislative amendments 4) Strengthening primary care workforce 5) Some forms of direct access model may already be in practice (though with questionable legal basis)
	Barriers	Regulatory gaps (e.g., lack of online physiotherapist directory showing their profiles to facilitate patients for seeking physiotherapy services, and presence of unregulated “pain management centres”)
Inner Setting *(Tension for change*,* Compatibility)*	Facilitators	Addressing shortage of doctors
	Barriers	Workforce constraints and service backlogs in public sector
Characteristics of individuals *(Individual stage of change*,* Knowledge & Beliefs about the Intervention*,* Self-efficacy)*	Facilitators	1) Formation of inter-disciplinary Working Group on Direct Access under the Physiotherapists Board of Hong Kong to prepare proposal for discussion 2) International recognition of the qualification and training received by physiotherapists in Hong Kong
	Barriers	Concerns on patient safety from doctors
(B) Policy design options		
1. Intervention characteristics *(Design quality & packaging*,* Adaptability*,* Complexity)* 2. Inner Setting *(Compatibility)* 3. Process *(Culture*,* Planning)*	Facilitators	Enhance communication and collaboration between doctors particularly doctors in public sector and private physiotherapists
	Barriers	*Condition 1: Patients can produce proof of diagnosis from a registered medical practitioner within the last 12 months* 1) Unclear definitions and insufficient details 2) Liability concerns related to proof of diagnosis (especially among doctors) 3) Not much difference from the current requirement of referral letters 4) 12-month validity period is too long (i.e., concern of changing conditions by doctors) *Condition 2: Compliance with clinical protocol or cross-disciplinary collaboration arrangement promulgated by authorized bodies* 1) Unclear definitions and details of protocol 2) Confusion over the term “protocol” 3) Viewed as too prescriptive by physiotherapists 4) The wording “*Compliance with*” seems to de-skill/ de-professionalise physiotherapists 5) Uncertainty about who to draft the protocols; time and resources burden 6) Operational complications and confusions among the providers *Condition 3: Emergencies and other circumstances endorsed by the Supplementary Medical Profession Council* 1) Already practising direct access in Residential Care Homes for Elderly (RCHEs) for elderly admission & referrals from Social Welfare Department/ Non-Governmental Organizations 2) Physiotherapists practise as a first aider in sports field currently 3) Difficulty defining all possible circumstances 4) Doctors perceived that only a few emergency situations require physiotherapy services
(C) Implementation		
Intervention characteristics *(Complexity*,* Trialability)*	Facilitators	1) Phased or pilot-based implementation approach to let buy-in before full-run (e.g., targeting specific conditions or settings) 2) Public education targeting low health literacy ones and chronic patients)
	Barriers	Complicated design and implementation: Confusion and different interpretations of the “proof of diagnosis” *(condition 1)* and “protocol-driven approach *(condition 2)*; and definition of the actual circumstances of “emergencies and certain other circumstances” *(condition 3)*
Outer Setting *(Patient Needs & Resources*,* External Policy & Incentives)*	Facilitators	1) Clear communication of policy goals 2) Public education 3) Legislative amendments and revisions in professional code of practice 4) Pre-defined governance framework to establish pathways or platforms to set clinical protocols
	Barriers	1) Patients unable to distinguish qualified physiotherapists 2) Limited public understanding of physiotherapy roles and direct access 3) Patients’ uncertainty about when to consult physiotherapists vs. doctor 4) Potential abuse of physiotherapy services perceived by insurers
Inner Setting *(Tension for change*,* Available resources*,* Compatibility*,* Access to Knowledge & Information*,* Implementation Climate)*	Facilitators	1) Other existing regulatory frameworks to supplement the regulation of physiotherapists (e.g., Private Healthcare Facilities Ordinance to regulate clinics) 2) Tertiary education institutions continuously updated teaching curriculum meeting international standards 3) Mandatory Continuous Professional Development 4) Retention efforts by the Hong Kong Hospital Authority to retain physiotherapists in public sector
	Barriers	1) Concerns about quality assurance, risk of misdiagnosis, and service abuse 2) Doctors’ concerns on physiotherapist competency and capability without a medical diagnosis 3) Limited readiness for information technology and infrastructure to support implementation 4) Concerns about the brain drain of physiotherapists from the public sector to the private sector
Characteristics of individuals *(Knowledge & beliefs about the intervention*, *individual stage of change)*	Facilitators	1) Inter- (or multi- / trans-) disciplinary education and engagement 2) Inputs from different stakeholders (e.g., doctors, physiotherapists, academics, patients) in the process 3) Training affirmed physiotherapists capabilities in establishing physical diagnosis, red flag screening and in clinical judgement 4) Competency in identifying red flags and making subsequent referrals
	Barriers	1) Insufficient understanding on the details of government proposal 2) Patients usually seek care from whoever they trust more 3) Insufficient interdisciplinary trust and coordination (Physiotherapist-doctors tension, professional boundaries)
Process of implementation *(Planning*,* Engaging*,* Executing*,* Reflecting & evaluating)*	Facilitators	1) Phased implementation with pilots for common conditions 2) Early and ongoing communication between government and stakeholders 3) Information and transparency through an online physiotherapist directory with physiotherapists’ profiles and mandatory electronic health record system sharing 4) Robust monitoring and regulation: complaint and disciplinary mechanism, random 5) Evaluation of impact after implementation
	Barriers	1) Inadequate engagement and consultation with different stakeholders 2) Limited tools for patient access and care coordination (online physiotherapist directory, electronic health record sharing systems) 3) Concern of patient privacy on patient information sharing 4) Concerns about quality assurance, complaint procedures, and risk of increased complaints 5) Lack of comprehensive information of physiotherapists to enable informed choice of patients

**Table 3:** Key informant interviews and focus group discussions example quotes

Citation	Quotes
(A) Perceived Need for the Direct Access Model	
1	*“So*,* as I mentioned earlier*,* over the past 10 years*,* there haven’t been any incidents. We’ve also conducted outcome measures and found that it has helped patients. There have been significant improvements in both pain and clinical outcomes.” (A representative from physiotherapy regulatory body)*
2	*“Taking my own example*,* spraining an ankle is a minor issue*,* but need to see a physiotherapist. I have to spend hundreds of dollars at a clinic just to get a referral letter… If I need a referral letter for every different pain*,* it would be very troublesome.” (A patient)*
3	*“From the medical perspectives*,* we absolutely support this access as it can reduce patient waiting times. The most important aspects are public interest and patient safety. We need to ensure that any new arrangement does not have negative impacts on these areas.” (A representative from a medical professional body)*
4	*“Part of our duty*,* of course*,* is to screen for red flag*,* and assess whether the patient is suitable for physiotherapy. If the patient has already seen a rheumatologist and has a treatment plan*,* we will conduct further assessment and use that information to provide appropriate care.” (A physiotherapist)*
(B) Policy Design Options	
5	*“… either we do nothing*,* or we move forward. But going backward is unacceptable. The three proposals the government put forward are not progress; not only do they not move forward*,* but they are regressions.” (A representative from academic institution)*
6	*“So*,* say the patient takes your note – proof of diagnosis – and later it turns out he actually had something serious*,* like a lung tumor. Then he comes back and says ‘You told me I only had arm pain that day*,*’ and uses that proof to sue you… So*,* I really wonder*,* is it necessary to have this kind of proof of diagnosis? And*,* if we must have it*,* is there a way to protect the doctor – like making it clear that this proof only reflects what was seen in that day*,* not a full diagnosis of everything.” (A doctor)*
7	*“From basic sciences like anatomy and physiology to understanding pathology*,* we need (our students) to understand different cases and health issues from a medical perspective. While our training is not the same as doctors*,* we have fundamental knowledge of pathology and medical conditions… Interprofessional education helps us understand when a condition is beyond our scope and when to refer patients appropriately.” (A representative from academic institution)*
8	*“Take chronic conditions like frozen shoulder*,* I think a period of 10 to 12 months is already quite generous. If you haven’t seen a doctor for the same issue in that time*,* and asked to go again*,* yes*,* it’s inconvenient and costs money. But I still think it’s worth (to go after 12 months)*,* just because you think it’s an old injury doesn’t mean it actually is. We’re just common people*,* not doctors. A lot of times*,* these things only get picked up during routine check-ups.” (A community patient)*
9	*“However*,* I also think about it from their [policymakers’] point of view*,* do they have the resources to provide the protocol for each and every pain or disease?” (A physiotherapist)*
10	*“…I spoke with a senior physiotherapist in sports. He said many physiotherapists on teams don’t display their credentials due to liability concerns in sports matches. Instead*,* they identify as first aiders because their first aid certification covers liability in emergencies. This regulation*,* as it stands*,* doesn’t offer much help. I’m not sure how they can define “emergency” in this context.” (A physiotherapist)*
11	*“I believe that if doctors and physiotherapists can engage in open dialogue and collaborate*,* with both parties willing to give way and find common ground*,* they can reach a shared understanding of what constitutes red flags. Establishing a standardised red flag list would improve clarity and consistency in implementation.” (A public sector doctor)*
(C) Implementation process	
12	*“Like I mentioned*,* it is about educating the public – helping them understand who to consult for which condition. It is not about giving strict instructions*,* but offering advice so patients can make better choices. I think that would really help*,* especially from the patient’s side.” (A community patient)*
13	*“Physiotherapists… they’re very knowledgeable about biology*,* about the body…I guess?…… But honestly*,* I don’t know what my options are*,* so I just call the ambulance. That’s it. I think the simplest kind of education is just letting the public know what steps they can take – what choices they have to deal with pain*,* or whatever situation they’re facing. That’s it. Just keep it simple.” (A community patient)*
14	*“Taking back pain - everyone says they have back pain or neck pain. These are the most commonly abused cases. I’ve seen situations where someone claimed back pain*,* but what they actually received was a sports massage*,* written up as physiotherapy. There’s a lot of this kind of misuse. People say they have foot pain*,* shoulder pain*,* and then go for ultrasounds or massages. It happens often. Even now*,* when we require a referral letter*,* we still see cases of abuse.” (A representative from an insurance agency)*
15	*“Once direct access is implemented*,* I will imagine it’ll be harder to hire physiotherapists. They’ll have more opportunities to open their own practices in the community*,* and take on private cases. Honestly*,* it’ll be more competitive to recruit a physiotherapist in our setting. But I see it this way - any government policy needs countermeasures. So*,* we’re considering separating part of our team or expanding to meet the rising demand for health services.” (A representative from service provider)*
16	*“Back in medical school*,* they mentioned ‘physiotherapy’*,* but we had no idea what it actually involved… When I was a houseman*,* my supervisor would say*,* ‘Refer this patient to physio*,*’ but we didn’t really know what physiotherapists did… Like*,* if someone had foot pain*,* we’d refer them to pain physiotherapy – but what is pain physiotherapy*,* really? We just didn’t know. So yes*,* improving mutual understanding and communication would definitely help.” (A private sector doctor)*
17	*“Instead of just having a registry*,* I actually think it would be more helpful if there was a system—like how we find general practitioners (GPs) now. GPs are everywhere*,* and it’s easy for the public to find one. But when it comes to physiotherapists*,* you don’t really see many standalone physiotherapy clinics on the street. That’s why I believe having a registry or an online system where people can search for physiotherapists’ addresses or other relevant information would be really helpful for the general public. …… I think a registry or an online platform would be quite effective in helping people access direct physiotherapy services without needing a referral.” (Patient)*

#### Perceived need for the direct access model

##### Intervention characteristics

There was broad consensus among participants regarding the necessity of a direct access model for physiotherapy services in Hong Kong. Under the CFIR domain of Intervention Characteristics, both physiotherapists and patients perceived the model as evidence-based, citing international precedents that demonstrated improved health outcomes, enhanced accessibility, cost-effectiveness, and minimal adverse events. Physiotherapists emphasized that their professional training aligns with international standards, reinforcing confidence in their capability to operate safely and effectively within a direct access model (Citation 1).

Some patients also highlighted the practical burdens of the current referral system, particularly the financial and logistical barriers to accessing care (Citation 2).

##### Outer setting

From the Outer Setting perspective, participants identified several systemic healthcare challenges - including an aging population, rising chronic disease burden, and long wait times in the public sector – as key drivers for reform. The direct access model was viewed as a potential strategy to enhance patient autonomy, reduce unnecessary bottlenecks in care pathways, and alleviate pressure on the medical workforce. These goals were seen as aligning with the government’s long-term primary care strategy as reported by the policymaker.

##### Inner setting

Within the Inner Setting, a tension for change was observed, particularly in the public sector where workforce constraints and service backlogs have created significant operational pressure. However, resistance from segments of the medical community – primarily relate to concerns about patient safety as the patient is commencing physical treatment without a definitive medical diagnosis with the potential of patients having some serious underlying problems, and the disruption of traditional doctor-led practice model - emerged as a notable barrier (Citation 3).

##### Characteristics of individuals

At the level of Characteristics of Individuals, the establishment of a working group under the Physiotherapists Board to develop reform proposals was seen as a proactive step. Physiotherapists also demonstrated their strong professional self-efficacy, expressing confidence in their ability to manage patients independently while recognizing the importance of interprofessional collaboration and appropriate referral when necessary (Citation 4).

#### Policy design options

The second theme centres on the design of proposed policy options for implementing a direct access model, as developed by the HKSAR Government [9, 10]. In line with the Chief Executive’s 2021 Policy Address advocating legislative reform to enable direct access to physiotherapy services [17], the Physiotherapists Board of Hong Kong convened an interdisciplinary Working Group on Direct Access [18]. This group, comprising doctors, physiotherapists, and community representatives, proposed a model allowing patients to access physiotherapy in private primary care settings without a doctor’s referral. The model included differentiated practice limits and timeframes for patients with or without pre-existing medical diagnoses [18]. In December 2023, the Government introduced an alternative model informed by stakeholder feedback. This version outlined three specific conditions under which direct access would be permitted: (a) the presence of a proof of diagnosis, (ii) compliance with clinical protocols, and (iii) clearly defined emergencies and other circumstances [9]. While this proposal outlines how a patient can seek help from a physiotherapist without going through a doctor when there is a definitive diagnosis, when there is no prior medical diagnosis, and under emergency conditions, the proposal created substantial concerns and lacked sufficient details for the understanding of their intent.

##### Concerns over policy design and clarity

The vast majority of focus group participants expressed concerns regarding the complexity and clarity of the government’s proposal (design quality and packaging aspect of CFIR). Stakeholders, particularly from the physiotherapy sector - including members of the Physiotherapists Board, academic institutions, and frontline physiotherapists - perceived the government’s model as a “setback” or a step backward compared to the Working Group’s original proposal. They questioned why the government did not directly adopt or respond to the Working’s Group’s recommendation (Citation 5).

A key point of contention was the requirement for patients to present a “proof of diagnosis” within the past 12 months. Physiotherapists criticized this criterion as ambiguous, noting that it closely resembled the existing referral system without offering meaningful reform. The doctors also raised concerns about the legal liability associated with such proof, noting that its legal standing - unlike a formal referral letter what constituted proof- was unclear and could lead to disputes (Citation 6).

While policymakers and doctors emphasized the need for a medical diagnosis to ensure patient safety, most physiotherapists disagreed with this requirement, viewing it as a barrier to true direct access. They argued that their training equips them to identify red flags in their assessment of patients and when it is necessary to refer the patients to doctors when necessary (Citation 7).

There were mixed views on the 12-month validity period for the proof of diagnosis. Some doctors expressed concerns that this period may be too long, as patients’ conditions could change significantly. In contrast, most physiotherapists and patients considered the timeframe appropriate, given the chronic nature of many musculoskeletal conditions (Citation 8).

##### Concerns about clinical protocols

The second condition - requiring compliance with clinical protocols - was viewed by physiotherapists as overly prescriptive and potentially de-skilling. They expressed concern that rigid protocols could undermine their clinical judgement and autonomy and limit their ability to tailor treatments needed by individual patients. Moreover, various stakeholders reported confusion over the definition and scope of “clinical protocols”, with varying interpretations across professions. Some physiotherapists suggested using the term clinical guidelines – rather than strict protocols - would be more appropriate, as guidelines recognized need for clinical judgement and offer flexibility while maintaining safety. However, doctors tended to be more conservative, preferring standardized protocols for each diagnosis, particularly when patient safety was a concern (Citation 9).

All participants agreed that further clarification and guidance were needed regarding protocol development, implementation and oversight. Physiotherapists and patients also questioned the feasibility of developing comprehensive protocols given the time and resources required.

##### Clear definition around emergencies and special circumstances

The third condition – definition of allowing direct access in emergencies or special circumstances endorsed by the Supplementary Medical Profession Council - elicited mixed reactions. Physiotherapists noted the difficulty of defining all possible emergencies and special circumstances for direct access. Indeed, it was reported that physiotherapists already provide care for patients admitted to the elderly homes, often without formal referrals.

Doctors and physiotherapists held divergent views on what constitutes an emergency. Doctors typically associated emergencies with acute, life-threatening conditions requiring attendances at Accident and Emergency departments. In contrast, physiotherapists highlighted their role in providing urgent care for musculoskeletal problems in sports and community settings. One physiotherapist noted that, due to liability concerns, they often avoid citing their professional title in such contexts (Citation 10).

##### Process and stakeholder engagement

Under the CFIR domain of Process, participants emphasized the need for improved planning, clear communication, and more inclusive engagement. Many believed that enhanced engagement and collaboration between doctors and physiotherapists would be essential to refining the policy design and ensuring successful implementations (Citation 11).

#### Implementation process

Findings related to implementation revealed both strategic opportunities and operational challenges across multiple CFIR domains.

Under Intervention Characteristics, a phased or pilot-based implementation approach was widely supported by participants. They suggested targeting specific conditions (e.g., low back pain, dementia) or settings (e.g., elderly residential care homes, sports institutes) to test feasibility and build stakeholder confidence. Such an approach was seen as a pragmatic way to manage risks and demonstrate early success.

Public education was also emphasized as critical enabler, particularly for populations with low health literacy. Participants noted that without adequate public awareness, patients may not understand when and how to access physiotherapy services directly (Citation 12).

From the Outer Setting perspective, key facilitators identified included the need for legislative amendments, clear articulation of policy goals, and establishment of a predefined governance framework. These were seen as essential to ensure accountability and consistency in implementation.

However, barriers were noted. Participants highlighted that many patients have limited understanding of the role of physiotherapists and may be uncertain when to seek physiotherapy versus medical care. This knowledge gap could hinder uptake of the direct access model (Citation 13).

Additionally, external factors such as the recognition of insurance reimbursement play a significant role in the successful implementation of direct access to physiotherapy services. Insurance agencies expressed concerns about potential overuse and abuse if patients are allowed to self-refer without a doctor’s referral. In response, some suggested implementing safeguards such as setting a ceiling on the number and cost of reimbursable physiotherapy visits or introducing a co-payment requirement.

However, one insurance agency noted that physiotherapy services constitute only a small proportion of overall claims, suggesting the unintended effects of direct access might be limited. Instead, the participant expressed greater concern about the complexity and resources required to verify the condition(s) eligible for reimbursement under a direct access model. This perspective was echoed by a service provider, who also highlighted the administrative burden such verification processes could impose (Citation 14).

Within the Inner Setting, several facilitators were noted, including the alignment of physiotherapy curricula with international standards, ongoing professional development, and potential application of existing regulatory frameworks, such as the Private Healthcare Facilities Ordinance to support the regulation of physiotherapy clinics.

Nonetheless, concerns persisted. Some stakeholders questioned the competency of physiotherapists to manage patients without a prior medical diagnosis, particularly in complex cases. Other raised concerns about the availability of resources to support implementation. Additionally, a few service providers expressed concern about a potential brain drain from the public to private sector, should direct access be implemented without adequate workforce planning (Citation 15).

In the domain of characteristics of individual level, interdisciplinary education and stakeholder engagement were seen as key enablers. Participants noted that shared training experiences and collaborative practice models could foster mutual understanding and trust between doctors and physiotherapists.

However, insufficient understanding of the government’s proposal and lingering trust issues between professional groups were identified as barriers. Some physiotherapists felt that their role was not fully recognized or respected by other healthcare professionals, which could hinder collaborative implementation (Citation 16).

Under the CFIR domain of Process, participants emphasized the importance of transparent communication, inclusive stakeholder consultation, and robust monitoring mechanisms. Several practical tools were proposed to support implementation, including an online physiotherapist directory showing their profiles to facilitate patients for seeking physiotherapy services and to improve public access, mandatory electronic health record sharing between providers, and regular audits to ensure quality and safety. However, barriers such as inadequate stakeholder engagement, lack of integrated patient information systems, and concerns about data privacy were also raised (Citation 17).

## Discussion

This study explored stakeholder perspectives on the policy design and implementation for a model of direct access to physiotherapy in Hong Kong, using the CFIR to identify key barriers and facilitators. While there was broad support for the model, particularly in addressing healthcare delivery system inefficiencies and improving patient access, several barriers identified were related to policy design, clarity, interprofessional dynamics and interactions, and system readiness. These findings of the study align with international experiences. In addition, the study findings offer additional context-specific insights for policy adaptation in traditionally physician-led healthcare systems.

### Facilitators for implementation

#### Evidence based and global precedents

Stakeholders widely acknowledged the strong emerging evidence base supporting direct access to physiotherapy particularly for musculoskeletal conditions. International studies have demonstrated that direct access can lead to improved clinical outcomes, reduced healthcare costs, and enhanced patient satisfaction, with no increase in adverse events [4, 14]. In this study, physiotherapists and patients viewed the model as evidence-based and aligned with international standards, reinforcing confidence in its safety and effectiveness.

#### Professional readiness and training

Physiotherapists and the training institutions expressed confidence in their training, particularly in screening for red flags and making appropriate referrals. This aligns with global findings that emphasize the importance of equipping physiotherapists with competencies in assessment and clinical judgement [4, 19, 20]. The establishment of a working group under the Physiotherapists Board and the alignment of local curricula with international standards were seen as important enablers of professional readiness for the implementation of the direct access model in Hong Kong.

#### Policy momentum and system alignment

The proposed reform was viewed as consistent with the government’s broader primary care strategy. Stakeholders noted that the policy could help alleviate pressure on the public healthcare system, reduce waiting times, and enhance patient access and choice. These views reflect international trends where direct access has been integrated into primary care reforms to improve system efficiency and responsiveness [7].

### Barriers in implementation

#### Ambiguity in policy design

A major barrier identified was the perceived conservatism and ambiguity in the government’s proposed model—particularly around the requirement for “proof of diagnosis,” the use of clinical protocols, and the definition of emergencies. Stakeholders perceived these conditions as overly complex and potentially regressive compared to the Working Group’s original proposal. Similar concerns have been reported in other jurisdictions, where unclear eligibility criteria and legal ambiguities have hindered implementation [21]. This speaks for clarity of the policy design during the formulation process, and closer communication and engagement during the implementation process.

#### Interprofessional tensions and trust

Resistance from segments of the medical community—particularly regarding patient safety and the disruption of traditional referral pathways—was a recurring theme. While some doctors supported the model in principle, others expressed concerns about physiotherapist competency in managing undiagnosed medical conditions. These findings echo the international literature which highlight interprofessional trust and role clarity as critical factors in the success of direct access models [15]. Breaking the silos between professions may help understanding each other’s role, and reassure the doctors about the refer-back mechanisms.

#### Public and patient awareness

Limited public understanding of physiotherapy roles and uncertainty about when to seek physiotherapy versus medical care were seen as barriers to uptake. Participants emphasized the need for public education campaigns to improve health literacy and support informed decision-making. This is consistent with global experiences, where public awareness has been a key determinant of successful implementation [22].

#### Insurance and financial structures

Concerns about potential overuse, abuse, and administrative burden were raised by insurance agencies in relation to a direct access model. While some insurers supported the model in principle, they emphasized the need for safeguards such as reimbursement ceilings, visit limits, or co-payment mechanisms to mitigate misuse. Insurers’ decision on reimbursement rules and claim requirement would influence provider behaviour and patient utilization patterns. For example, although Medicare in the United States removed the requirement for beneficiaries to obtain a physician referral in 2005, it still requires physician certification of physiotherapy plans of care, even under direct access. This requirement might constrain access to allied health services [23]. These findings indicate that clear financial policies are essential to enable that direct access to physiotherapy is feasible and sustainable in real-word implementation.

### Policy and practice implications

This study provides new insights of the roles of insurance agencies and financial mechanisms in facilitating the uptake of direct access in the implementation—an area underexplored the in existing literature. It is necessary to engage insurers early to co-design policies which include reimbursement safeguards and reduce administrative burden.

It also highlights the nuanced perspectives of doctors and physiotherapists, demonstrating that support or resistance arising from misunderstanding of respective roles may vary by specialty and clinical setting. Therefore, it is important to foster interprofessional collaboration through joint training and stakeholder engagement. Additionally, investing in public education to improve awareness of physiotherapy roles (such as restoring movement and function, preventing disability, and supporting long-term condition management) [24] and better clarification of policy scope, more details of the policy design and eligibility criteria is crucial to reduce ambiguity and legal uncertainty. Emphasis on phased implementation and pilot testing in specific conditions (e.g., musculoskeletal disorders), and contexts (e.g., elderly care) offers a pragmatic strategy for managing risk and building stakeholder confidence. Importantly, clear care escalation pathways should be included in the direct access model to ensure that if symptoms do not improve at the primary care level, timely referral to other healthcare professionals is facilitated.

A critical first step in this study is the development of an acceptable intervention design of the policy from the options considered. The design will define the intervention characteristics (CFIR’s domain), which has significant influence on the implementation factors - barriers and facilitators – across the other four CFIR domains. By integrating the implementation science approach with a subsequent deliberative process to facilitate stakeholder expert consensus in a Delphi Survey, this study advances the evidence base for designing acceptable, evidence-informed policy interventions and implementation strategies.

### Limitations and future research

This study included a limited number of insurance stakeholders, although those interviewed represented major providers. Future research should explore the perspectives of other allied health professionals who may be affected by similar policy changes. Additionally, while this study focused on stakeholder perceptions, future work should evaluate patient outcomes, cost-effectiveness, and service utilization following implementation.

## Conclusion

While there is broad support for direct access to physiotherapy at primary care level in Hong Kong, successful implementation will require careful attention to the policy design, system readiness, stakeholder alignment, and adaptive strategies that recognize professional judgement and autonomy while safeguarding patient safety. Lessons from global experiences reinforce the importance of evidence-informed policy design, collaborative governance, and continuous evaluation. These findings provide a foundation for developing context-specific implementation strategies, which can be further refined in a deliberative process to facilitate expert consensus in a subsequent Delphi study. Ultimately, our study aims to facilitate access to physiotherapy services in Hong Kong by integrating research insights into an acceptable policy design and actionable strategies for policy implementation with expert and stakeholder inputs.

## Supplementary Information

Supplementary Material 1.

Supplementary Material 2.

Supplementary Material 3.

## Data Availability

No datasets were generated or analysed during the current study.

## Abbreviations

CFIR: Consolidated Framework for Implementation Research
