# Supplementary material for: Barriers and facilitators of direct access model to physiotherapy: intervention design and implementation strategies using the consolidated framework for implementation research
Source: Arch Public Health. 2026 Mar 12;84:80. doi: 10.1186/s13690-026-01882-7 (PMC13094042; doi:10.1186/s13690-026-01882-7)
Supplement: Supplementary file 1 — Supplementary Material 1. [file 13690_2026_1882_MOESM1_ESM.pdf]

Development of the Implementation Strategies for the Policy of Direct Access (Patient Self-Referral) to Physiotherapists in Primary Care -

Discussion Guide (Taking reference to Consolidated Framework for Implementation Research)

For Representatives from Government Policy Decision Makers

Background

Both the Chief Executive's Policy Address 2021 and 2022 (Para 135 and Para 82 respectively) specified the need to allow the public to access direct services provided by physiotherapists without a doctor's referral, as part of the initiatives to strengthen primary healthcare.

Based on the issues raised in the Policy Address, the Working Group on Implementation of Modified Referral System for PT Services (the Working Group) under the Physiotherapists Board (PT Board) started to formulate the direct access model for implementation in October 2021. A proposal on "Direct Access Model for Physiotherapy Service in Hong Kong" was compiled by the Working Group which was then submitted to the PT Board in September 2022 and the Supplementary Medical Professions Council (SMPC) on 1 February 2023 for discussion and endorsement. The SMP Council wrote to the HHB in February 2023, summarising its members' views on the direct access arrangement for PTs while enclosing the proposal that the working group under the PT Board submitted to the SMP Council. Taking into account the suggestions of the SMP Council and the earlier comments made by various healthcare professions, the Government proposes exploring to amend the Ordinance to enable PTs and OTs to provide services directly to patients under specified circumstances without a doctor's referral including (1) only patients with a condition previously diagnosed by a doctor within a pre-defined period of time; (2) a protocol-driven approach; and (3) patients under emergency or institutional settings where doctors' referral may not be practical (e.g. services at residential care homes for the elderly or people with disabilities, special schools etc.).

(A) Views on PT Direct Access in Hong Kong

1. Do you think there is a need for direct access to services of physiotherapists in Hong Kong? Why? *(Intervention characteristics)*

2. What kind of supporting evidence are there about the effectiveness of direct access to physiotherapist? *(Intervention characteristics)*

3. Do you think the PT direct access will work in Hong Kong? *(Intervention characteristics)*

(B) Design of the Proposed Direct Access Model

4. How were the features of the proposed direct access model designed as mentioned in the latest discussion paper in the Legislative Council Panel on Health Services on 8 December 2023? Any reference? *(Intervention characteristics)*

   Probe:
   - *Patients can produce proof of diagnosis from a registered medical practitioner within the last 12 months*
   - *Compliance with clinical protocol or cross-disciplinary collaboration arrangement promulgated by authorised bodies*
   - *Provide emergencies and other circumstances endorsed by the SMP Council*

5. How does the direct access model proposed by SMP Council in the updated paper discussed at the Legislative Council Panel compared with the one developed by the Working Group previously and with other programs overseas? Advantage and disadvantage? *(Intervention characteristics & Outer setting)*

6. How well do you think the proposed direct access model can be adapted in Hong Kong to meet local needs? *(Intervention characteristics)*
   - Any features in the proposed direct access model that will facilitate adoption/ implementation? *(Intervention characteristics)*
   - What kind of changes need to be further made to the design of the proposed direct access model to facilitate implementation/ adaptation? i.e. parameters/ components for direct access? *(Intervention characteristics)*

(C) **Implementation Process**

7. Who will lead /take part in the implementation and execution of the direct access model? What will be the role of Government in this process? *(Process)*

8. Is there an implementation schedule? Will the proposed direct access model be piloted prior to full-scale implementation and how will it be piloted? *(Intervention characteristics)* Can you describe the plan for implementation? *(Process)*

9. What have been done (or what is planned to do) by the Government as preparation to facilitate implementation? *(Process)*

10. What kind of changes and support are required/ anticipated to facilitate adaptation? What kind of support was given / changes were made so far? *(Inner setting)*

    Probe:
    - *Changes to PT Board and SMPC*
    - *Legislative amendments*
    - *Code of Practice*

- *Clinical governance*
- *Availability of guideline for referral*
- *Public education / Information and materials about direct access to services of physiotherapists are made available to different stakeholders*

11. What are the costs anticipated to be incurred in implementing the direct access model? How do you expect to procure necessary resources? *(Intervention characteristics)*

    Probe:
    - *Funding models*
    - *Manpower*

12. How well/ ready do you think other stakeholders (i.e. public, PT, other healthcare professionals, employers) will adapt to PT direct access? Any barriers anticipated? *(Characteristics of individual)*

    Probe:
    - *Public knowledge and health-seeking behaviour*
    - *Skills and training of PT*
    - *Clarity over the role*
    - *Inter-professional collaborations*

13. What kinds of incentives are there to engage stakeholders and help ensure that the implementation of the direct access model is successful? *(Inner setting)*

14. Is there consensus with the proposed direct access model among key stakeholders i.e. features, implementation process? *(Inner setting)*

(D) Impact of Direct Access to PT Services

15. What is the extent of impact of PT Direct Access Model (Intervention goals)? *(Implementation, Services and Patient Outcome)*

    Probe:
    - Patient safety & quality of service
    - Availability/ accessibility
    - Efficiency / timeliness
    - Cost effectiveness
    - Accountability & transparency
    - Manpower planning

16. How will you assess progress towards implementation and intervention goals? *(Process, Implementation Outcome)* What will be assessed in the process of implementation (Implementation goals)?

    Probe:

- *Acceptability*
- *Adoption*
- *Appropriateness*
- *Feasibility*
- *Implementation cost*
- *Penetration*
- *Sustainability*

(E) Policy direction/ plan

17. How well do you think direct access to services of physiotherapists will fit into Government's long term goal on healthcare manpower planning and the role and professional development of allied health professionals in primary care? *(Outer setting)*

18. What other policies are needed to support the primary care development in Hong Kong? *(Outer setting)*

Development of the Implementation Strategies for the Policy of Direct Access (Patient Self-Referral) to Physiotherapists in Primary Care -

Discussion Guide (Taking reference to Consolidated Framework for Implementation Research)

For Representatives from Medical College

Background

Both the Chief Executive's Policy Address 2021 and 2022 (Para 135 and Para 82 respectively) specified the need to allow the public to access direct services provided by physiotherapists without a doctor's referral, as part of the initiatives to strengthen primary healthcare.

Based on the issues raised in the Policy Address, the Working Group on Implementation of Modified Referral System for PT Services (the Working Group) under the Physiotherapists Board (PT Board) started to formulate the direct access model for implementation in October 2021. A proposal on "Direct Access Model for Physiotherapy Service in Hong Kong" was compiled by the Working Group which was then submitted to the PT Board in September 2022 and the Supplementary Medical Professions Council (SMPC) on 1 February 2023 for discussion and endorsement. The SMP Council wrote to the HHB in February 2023, summarising its members' views on the direct access arrangement for PTs while enclosing the proposal that the working group under the PT Board submitted to the SMP Council. Taking into account the suggestions of the SMP Council and the earlier comments made by various healthcare professions, the Government proposes exploring to amend the Ordinance to enable PTs and OTs to provide services directly to patients under specified circumstances without a doctor's referral including (1) only patients with a condition previously diagnosed by a doctor within a pre-defined period of time; (2) a protocol-driven approach; and (3) patients under emergency or institutional settings where doctors' referral may not be practical (e.g. services at residential care homes for the elderly or people with disabilities, special schools etc.).

(A) Views on PT Direct Access in Hong Kong

1. Do you think there is a need for direct access to services of physiotherapists in Hong Kong? Why? *(Intervention characteristics)*

2. What kind of supporting evidence are there about the effectiveness of direct access to physiotherapist? *(Intervention characteristics)*

3. Do you think the PT direct access will work in Hong Kong? *(Intervention characteristics)*

(B) Design of Direct Access Model

4. What are your views on the scope and conditions for allowing direct access to services of physiotherapists in Hong Kong? *(Intervention characteristics)*

   Probe:
   - *Settings*
   - *Patients can produce proof of diagnosis from a registered medical practitioner within the last 12 months*
   - *Compliance with clinical protocol or cross-disciplinary collaboration arrangement promulgated by authorised bodies*
   - *Provide emergencies and other circumstances endorsed by the SMP Council*
   - *Pre-requisite requirement/ competency of a physiotherapist conducting direct access including Mandatory continuing professional development*
   - *Insurance*

5. How well do you think the proposed direct access model can be adapted in Hong Kong to meet local needs? *(Intervention characteristics)*
   - Any features in the proposed direct access model that will facilitate adoption/ implementation? *(Intervention characteristics)*
   - What kind of changes need to be made to the design of the proposed direct access model to facilitate implementation/ adaptation? i.e. parameters/ components for direct access? *(Intervention characteristics)*

(C) Implementation Process

6. What kind of changes and support are necessary/ anticipated to facilitate adaptation? *(Inner setting)*

   Probe:
   - *Changes to PT Board and SMPC*
   - *Legislative amendments*
   - *Code of Practice*
   - *Clinical governance*
   - *Availability of guideline for referral*
   - *Incentives to engage different stakeholders*
   - *Public education / Information and materials about direct access to services of physiotherapists are made available to different stakeholders*

7. How receptive do you think your profession is to adapt to PT direct access? *(Inner setting)*

   Probe:
   - *Tension for change, i.e. Is there a need of the patients?*
   - *Compatibility, i.e. how well does direct access fit with existing work processes and practices?*

- *Leadership*
- *Organisational culture*
- *Clarity over the role*
- *Inter-professional collaborations*

8. How do you think the health seeking behaviour/ culture will affect the implementation of the PT direct access? *(Inner setting)* Specifically any kind of incentives and support are necessary to engage patients to self-refer to physiotherapy services? *(Inner setting)*

9. How well/ ready do you think other stakeholders (i.e. PTs, other healthcare professionals, employers) will adapt to PT direct access? Any barriers anticipated? *(Outer setting, Characteristics of individual)*

10. Is there consensus with the proposed direct access model among key stakeholders i.e. features, implementation process? *(Inner setting)*

11. How complicated is it to execute PT direct access in Hong Kong? Any facilitators or barriers anticipated? *(Intervention characteristics)*

(D) Impact of Direct Access to PT Services

12. What is the extent of impact of PT Direct Access Model (Intervention goals)? *(Implementation Outcome)*

    Probe:
    - Patient safety & quality of service
    - Availability/ accessibility
    - Efficiency / timeliness
    - Cost effectiveness
    - Accountability & transparency
    - Manpower planning

Development of the Implementation Strategies for the Policy of Direct Access (Patient Self-Referral) to Physiotherapists in Primary Care -

Discussion Guide (Taking reference to Consolidated Framework for Implementation Research)

For Representatives from Physiotherapists Board

Background

Both the Chief Executive’s Policy Address 2021 and 2022 (Para 135 and Para 82 respectively) specified the need to allow the public to access direct services provided by physiotherapists without a doctor’s referral, as part of the initiatives to strengthen primary healthcare.

Based on the issues raised in the Policy Address, in October 2021, the Working Group on Implementation of Modified Referral System for PT Services (WG) under the Physiotherapists Board (PT Board) formulated a proposal on “Direct Access Model for Physiotherapy Service in Hong Kong”, and submitted to the Supplementary Medical Professions Council (SMP) Council for discussion and endorsement in February 2023. The SMP Council wrote to the HHB in February 2023, summarising its members’ views on the direct access arrangement for PTs while enclosing the proposal that the working group under the PT Board submitted to the SMP Council. Taking into account the suggestions of the SMP Council and the earlier comments made by various healthcare professions, the Government proposes exploring to amend the Ordinance to enable PTs and OTs to provide services directly to patients under specified circumstances without a doctor’s referral including (1) only patients with a condition previously diagnosed by a doctor within a pre-defined period of time; (2) a protocol-driven approach; and (3) patients under emergency or institutional settings where doctors’ referral may not be practical (e.g. services at residential care homes for the elderly or people with disabilities, special schools etc.).

(A) Views on PT Direct Access in Hong Kong

1. Do you think there is a need for direct access to services of physiotherapists in Hong Kong? Why? *(Intervention characteristics)*

2. What kind of supporting evidence are there about the effectiveness of direct access to physiotherapist? *(Intervention characteristics)*

3. Do you think the PT direct access will work in Hong Kong? *(Intervention characteristics)*

(B) Design of the Proposed Direct Access Model by WG and the Government

4. Can you describe the development process of the WG's proposal of the direct access model, i.e. timeline, views of stakeholders, consensus building, and amendments made in the process? *(Process) (inner setting)*

   Probe:
   - *How long was the development process? How many meetings were arranged?*
   - *How were the features of the WG's proposed direct access model designed e.g. settings, clients, pre-requisite requirement, exemption, insurance? Any reference? (Intervention characteristics)*
   - *Were the views of all concerned stakeholders represented? How were the opinions from concerned stakeholders collected? What are the major concerns?*
   - *Was consensus reached within the working group? Is there a consensus building process?*
   - *What kind of changes were made to the proposed direct access model to facilitate implementation, e.g. additional measures to reach consensus among the working group?*

5. To what extent were the needs and preferences of the public considered when designing the direct access model? *(Outer setting)*

6. How does the direct access model in the updated paper discussed at the Legislative Council Panel on 8 December 2023 compared with the one developed by the Working Group previously and with other programs overseas? Advantage and disadvantage? *(Intervention characteristics)*

   Probe: [Discussion Paper at Legislative Council Panel]
   - *Patients can produce proof of diagnosis from a registered medical practitioner within the last 12 months*
   - *Compliance with clinical protocol or cross-disciplinary collaboration arrangement promulgated by authorised bodies*
   - *Provide emergencies and other circumstances endorsed by the SMP Council*

7. How well do you think the proposed direct access model can be adapted in Hong Kong to meet local needs? *(Intervention characteristics)*
   - Any features in the proposed direct access model that will facilitate adoption/ implementation? *(Intervention characteristics)*
   - What kind of changes need to be made to the design of the proposed direct access model to facilitate implementation/ adaptation? i.e. parameters/ components for direct access? *(Intervention characteristics)*

(C) **Implementation Process**

8. Who will lead / take part in the implementation and execution of the direct access model? What will be the role of PT Board in this process? *(Process)*

9. Is there an implementation schedule? Will the proposed direct access model be piloted prior to full-scale implementation and how will it be piloted? (Intervention characteristics) Can you describe the plan for implementation? (Process)

10. What kind of changes and support are necessary/ anticipated to facilitate adaption? What kind of support was given / changes were made so far? *(Intervention characteristics, , Inner setting)*

    Probe:
    - *Changes to PT Board and SMPC*
    - *Legislative amendments*
    - *Code of Practice*
    - *Clinical governance*
    - *Availability of guideline for referral*
    - *Incentives to engage different stakeholders*
    - *Public education / Information and materials about direct access to services of physiotherapists are made available to different stakeholders*

11. What have been done (or what is planned to do) as preparation to facilitate implementation? *(Process)*

12. How do you think the health seeking behaviour/ culture will affect the implementation of the PT direct access? *(Inner setting)*

13. How well/ ready do you think other stakeholders (i.e. public, PTs, other healthcare professionals, employers) will adapt to PT direct access? Any barriers anticipated? *(Outer setting)*

    Probe:
    - Public knowledge and health-seeking behaviour
    - Skills and training of PTs
    - Clarity over the role
    - Inter-professional collaborations

14. What level of involvement/ leadership was observed & what kind of support was observed from different stakeholders so far with implementing the direct access model? *(Inner setting)*

15. How complicated is it to execute the proposed direct access model? Any facilitators or barriers anticipated? *(Intervention characteristics)*

(D) Impact of Direct Access to PT Services

16. What is the extent of impact of PT Direct Access Model (Intervention goals)? *(Implementation Outcome)*

Probe:
- Patient safety & quality of service
- Availability/ accessibility
- Efficiency / timeliness
- Cost effectiveness
- Accountability & transparency
- Manpower planning

17. How will you assess progress towards implementation and intervention goals? *(Process, Implementation Outcome)* What will be assessed in the process of implementation (Implementation goals)? *(Process)*

    Probe:
    - *Acceptability*
    - *Adoption*
    - *Appropriateness*
    - *Feasibility*
    - *Implementation cost*
    - *Penetration*
    - *Sustainability*

Development of the Implementation Strategies for the Policy of Direct Access (Patient Self-Referral) to Physiotherapists in Primary Care -

Discussion Guide (Taking reference to Consolidated Framework for Implementation Research)

For Representatives from Physiotherapy Industry

Background

Both the Chief Executive's Policy Address 2021 and 2022 (Para 135 and Para 82 respectively) specified the need to allow the public to access direct services provided by physiotherapists without a doctor's referral, as part of the initiatives to strengthen primary healthcare.

Based on the issues raised in the Policy Address, the Working Group on Implementation of Modified Referral System for PT Services (the Working Group) under the Physiotherapists Board (PT Board) started to formulate the direct access model for implementation in October 2021. A proposal on "Direct Access Model for Physiotherapy Service in Hong Kong" was compiled by the Working Group which was then submitted to the PT Board in September 2022 and the Supplementary Medical Professions Council (SMPC) on 1 February 2023 for discussion and endorsement. The SMP Council wrote to the HHB in February 2023, summarising its members' views on the direct access arrangement for PTs while enclosing the proposal that the working group under the PT Board submitted to the SMP Council. Taking into account the suggestions of the SMP Council and the earlier comments made by various healthcare professions, the Government proposes exploring to amend the Ordinance to enable PTs and OTs to provide services directly to patients under specified circumstances without a doctor's referral including (1) only patients with a condition previously diagnosed by a doctor within a pre-defined period of time; (2) a protocol-driven approach; and (3) patients under emergency or institutional settings where doctors' referral may not be practical (e.g. services at residential care homes for the elderly or people with disabilities, special schools etc.).

(A) Views on PT Direct Access in Hong Kong

1. Do you think there is a need for direct access to services of physiotherapists in Hong Kong? Why? *(Intervention characteristics)*

2. What kind of supporting evidence are there about the effectiveness of direct access to physiotherapist? *(Intervention characteristics)*

3. Do you think the PT direct access will work in Hong Kong? *(Intervention characteristics)*

(B) Design of Direct Access Model

4. What are your views on the scope and conditions for allowing direct access to services of physiotherapists in Hong Kong? *(Intervention characteristics)*

   Probe:
   - *Settings*
   - *Patients can produce proof of diagnosis from a registered medical practitioner within the last 12 months*
   - *Compliance with clinical protocol or cross-disciplinary collaboration arrangement promulgated by authorised bodies*
   - *Provide emergencies and other circumstances endorsed by the SMP Council*
   - *Pre-requisite requirement/ competency of a physiotherapist conducting direct access including Mandatory continuing professional development*
   - *Insurance*

5. How well do you think the proposed direct access model can be adapted in Hong Kong to meet local needs? *(Intervention characteristics)*
   - Any features in the proposed direct access model that will facilitate adoption/ implementation? *(Intervention characteristics)*
   - What kind of changes need to be made to the design of the proposed direct access model to facilitate implementation/ adaptation? i.e. parameters/ components for direct access? *(Intervention characteristics)*

(C) Implementation Process

6. What kind of changes and support are necessary/ anticipated to facilitate adaptation? *(Inner setting)*

   Probe:
   - *Changes to PT Board and SMPC*
   - *Legislative amendments*
   - *Code of Practice*
   - *Incentives to engage different stakeholders*
   - *Public education / Information and materials about direct access to services of physiotherapists are made available to different stakeholders*

7. How receptive do you think the PT industry is to adapt to PT direct access? *(Inner setting)*

   Probe:
   - *Tension for change, i.e. Is there a need of the patients?*
   - *Compatibility, i.e. how well does direct access fit with existing work processes and practices?*
   - *Leadership*
   - *Organisational culture*
   - *Clarity over the role*

- *Inter-professional collaborations*

8. How confident do you feel your profession has the capacity and capability to implement PT direct access? Is your profession equipped with the knowledge/ training that can form the basis for direct access? *(Characteristics of individual)*

9. How well/ ready do you think other stakeholders (i.e. public, other healthcare professionals, employers) will adapt to PT direct access? Any barriers anticipated? *(Characteristics of individual)*

   Probe:
   - Public needs and knowledge, health seeking behaviour
   - Clarity over the role
   - Inter-professional collaborations

10. How complicated is it to execute direct access to services of physiotherapists in Hong Kong? Any facilitators or barriers anticipated? *(Intervention characteristics)*

(D) Impact of Direct Access to PT Services

11. What is the extent of impact of PT Direct Access Model (Intervention goals)? *(Implementation Outcome)*

   Probe:
   - Patient safety & quality of service
   - Availability/ accessibility
   - Efficiency / timeliness
   - Cost effectiveness
   - Accountability & transparency
   - Manpower planning

Development of the Implementation Strategies for the Policy of Direct Access (Patient Self-Referral) to Physiotherapists in Primary Care - Discussion Guide (Taking reference to Consolidated Framework for Implementation Research)

For Representatives from Employers/ Provider Organisations/ NGOs

Background

Both the Chief Executive's Policy Address 2021 and 2022 (Para 135 and Para 82 respectively) specified the need to allow the public to access direct services provided by physiotherapists without a doctor's referral, as part of the initiatives to strengthen primary healthcare.

Based on the issues raised in the Policy Address, in October 2021, the Working Group on Implementation of Modified Referral System for PT Services (WG) under the Physiotherapists Board (PT Board) formulated a proposal on "Direct Access Model for Physiotherapy Service in Hong Kong", and submitted to the Supplementary Medical Professions Council (SMP) Council for discussion and endorsement in February 2023. The SMP Council wrote to the HHB in February 2023, summarising its members' views on the direct access arrangement for PTs while enclosing the proposal that the working group under the PT Board submitted to the SMP Council. Taking into account the suggestions of the SMP Council and the earlier comments made by various healthcare professions, the Government proposes exploring to amend the Ordinance to enable PTs and OTs to provide services directly to patients under specified circumstances without a doctor's referral including (1) only patients with a condition previously diagnosed by a doctor within a pre-defined period of time; (2) a protocol-driven approach; and (3) patients under emergency or institutional settings where doctors' referral may not be practical (e.g. services at residential care homes for the elderly or people with disabilities, special schools etc.).

(A) Views on PT Direct Access in Hong Kong

1. Do you think there is a need for direct access to services of physiotherapists in Hong Kong? Why? *(Intervention characteristics)*

2. What kind of supporting evidence are there about the effectiveness of direct access to physiotherapist? *(Intervention characteristics)*

3. Do you think the PT direct access will work in Hong Kong? *(Intervention characteristics)*

(B) Design of Direct Access Model

4. What are your views on the scope and conditions for allowing direct access to services of physiotherapists in Hong Kong? Any safeguard measures? *(Intervention characteristics)*

   Probe:
   - *Settings*
   - *Patients can produce proof of diagnosis from a registered medical practitioner within the last 12 months*
   - *Compliance with clinical protocol or cross-disciplinary collaboration arrangement promulgated by authorised bodies*
   - *Provide emergencies and other circumstances endorsed by the SMP Council*
   - *Insurance*

5. How well do you think the proposed direct access model can be adapted in Hong Kong to meet local needs? *(Intervention characteristics)*
   - Any features in the proposed direct access model that will facilitate adoption/ implementation? *(Intervention characteristics)*
   - What kind of changes need to be made to the design of the proposed direct access model to facilitate implementation/ adaptation? i.e. parameters/ components for direct access? *(Intervention characteristics)*

(C) Implementation Process

6. How receptive do you think your organisation is to adapt to PT direct access? *(Inner setting)*

   Probe:
   - *Tension for change, i.e. Is there a need of the patients or to meet organizational goals and objectives?*
   - *Compatibility, i.e. how well does direct access fit with existing work processes and practices in your setting?*
   - *Organisational culture*
   - *Clarity over the role*
   - *Inter-professional collaborations*

7. What kind of changes and support are necessary/ anticipated to facilitate implementation at your organisation or at system level? *(Inner setting)*

   Probe:
   Your organisation:
   - *Training for staff*
   - *Staffing and workload management*
   - *Support from management*
   - *Clinical governance*
   - *Availability of guideline for referral*
   - *Service monitoring*

At system level

- *Legislative amendments*
- *Code of Practice*
- *Incentives to engage different stakeholders*
- *Public education / Information and materials about direct access to services of physiotherapists are made available to different stakeholders*

8. How confident are you to implement PT direct access at your setting? E.g. Is your staff equipped with the knowledge/ training that can form the basis for direct access *(Characteristics of individual)*

9. How will you engage patients to self-refer to physiotherapy services? *(Process)*

10. How do you think the health seeking behaviour/ culture will affect the implementation of the PT direct access? *(Inner setting)*

11. How well/ ready do you think other stakeholders (i.e. public, PT, other healthcare professionals) will adapt to PT direct access? Any barriers anticipated? *(Characteristics of individual)*

    Probe:
    - Public needs and knowledge, health seeking behaviour
    - Competency of PT (knowledge and training)
    - Clarity over the role
    - Inter-professional collaborations

12. How complicated is it to execute PT direct access in Hong Kong? Any facilitators or barriers anticipated? *(Intervention characteristics)*

(D) Impact of Direct Access to PT Services

13. What is the extent of impact of PT Direct Access Model (Intervention goals)? *(Implementation Outcome)*

    Probe:
    - Patient safety & quality of service
    - Availability/ accessibility
    - Efficiency / timeliness
    - Cost effectiveness
    - Accountability & transparency
    - Manpower planning

Development of the Implementation Strategies for the Policy of Direct Access (Patient Self-Referral) to Physiotherapists in Primary Care -

Discussion Guide (Taking reference to Consolidated Framework for Implementation Research)

For Representatives from Insurance Agencies

Background

Both the Chief Executive's Policy Address 2021 and 2022 (Para 135 and Para 82 respectively) specified the need to allow the public to access direct services provided by physiotherapists without a doctor's referral, as part of the initiatives to strengthen primary healthcare.

Based on the issues raised in the Policy Address, in October 2021, the Working Group on Implementation of Modified Referral System for PT Services (WG) under the Physiotherapists Board (PT Board) formulated a proposal on "Direct Access Model for Physiotherapy Service in Hong Kong", and submitted to the Supplementary Medical Professions Council (SMP) Council for discussion and endorsement in February 2023. The SMP Council wrote to the HHB in February 2023, summarising its members' views on the direct access arrangement for PTs while enclosing the proposal that the working group under the PT Board submitted to the SMP Council. Taking into account the suggestions of the SMP Council and the earlier comments made by various healthcare professions, the Government proposes exploring to amend the Ordinance to enable PTs and OTs to provide services directly to patients under specified circumstances without a doctor's referral including (1) only patients with a condition previously diagnosed by a doctor within a pre-defined period of time; (2) a protocol-driven approach; and (3) patients under emergency or institutional settings where doctors' referral may not be practical (e.g. services at residential care homes for the elderly or people with disabilities, special schools etc.).

(A) Views on PT Direct Access in Hong Kong

1. Do you think there is a need for direct access to services of physiotherapists in Hong Kong? Why? *(Intervention characteristics)*

2. What kind of supporting evidence are there about the effectiveness of direct access to physiotherapist? *(Intervention characteristics)*

3. Do you think the PT direct access will work in Hong Kong? *(Intervention characteristics)*

(B) Design of Direct Access Model

4. What are your views on the scope and conditions for allowing direct access to services of physiotherapists in Hong Kong? *(Intervention characteristics)*

   Probe:
   - *Settings*
   - *Patients can produce proof of diagnosis from a registered medical practitioner within the last 12 months*
   - *Compliance with clinical protocol or cross-disciplinary collaboration arrangement promulgated by authorised bodies*
   - *Provide emergencies and other circumstances endorsed by the SMP Council*
   - *Pre-requisite requirement/ competency of a physiotherapist conducting direct access including Mandatory continuing professional development*
   - *Insurance*

5. How well do you think the proposed direct access model can be adapted in Hong Kong to meet local needs? *(Intervention characteristics)*
   - Any features in the proposed direct access model that will facilitate adoption/ implementation? *(Intervention characteristics)*
   - What kind of changes need to be made to the design of the proposed direct access model to facilitate implementation/ adaptation? i.e. parameters/ components for direct access? *(Intervention characteristics)*

(C) Implementation Process

6. What kind of changes and support are necessary/ anticipated to facilitate implementation at your insurance company or at system level? *(Inner setting)*

   Probe:
   Your insurance company
   - *Policy change, i.e. clauses for medical coverage, professional liability insurance*

   At system level
   - *Legislative amendments*
   - *Code of Practice*
   - *Availability of guideline for referral*
   - *Incentives to engage different stakeholders*
   - *Public education / Information and materials about direct access to services of physiotherapists are made available to different stakeholders*

7. How do you think the health seeking behaviour/ culture will affect the implementation of the PT direct access? *(Inner setting)*

8. How well/ ready do you think other stakeholders (i.e. PTs, other healthcare professionals, employers) will adapt to PT direct access? Any barriers anticipated? *(Characteristics of individual)*

Probe:
- Public needs and knowledge, health seeking behaviour
- Skills and training of PTs
- Clarity over the role
- Inter-professional collaborations

(D) Impact of Direct Access to PT Services

9. What is the extent of impact of PT Direct Access Model (Intervention goals)? *(Implementation Outcome)*

   Probe:
   - Patient safety & quality of service (Increased risk?)
   - Availability/ accessibility
   - Efficiency / timeliness
   - Cost effectiveness
   - Accountability & transparency
   - Manpower planning

Development of the Implementation Strategies for the Policy of Direct Access (Patient Self-Referral) to Physiotherapists in Primary Care - Discussion Guide (Taking reference to Consolidated Framework for Implementation Research)

For Representatives from Tertiary Education Institute offering Physiotherapy Program

Background

Both the Chief Executive's Policy Address 2021 and 2022 (Para 135 and Para 82 respectively) specified the need to allow the public to access direct services provided by physiotherapists without a doctor's referral, as part of the initiatives to strengthen primary healthcare.

Based on the issues raised in the Policy Address, in October 2021, the Working Group on Implementation of Modified Referral System for PT Services (WG) under the Physiotherapists Board (PT Board) formulated a proposal on "Direct Access Model for Physiotherapy Service in Hong Kong", and submitted to the Supplementary Medical Professions Council (SMP) Council for discussion and endorsement in February 2023. The SMP Council wrote to the HHB in February 2023, summarising its members' views on the direct access arrangement for PTs while enclosing the proposal that the working group under the PT Board submitted to the SMP Council. Taking into account the suggestions of the SMP Council and the earlier comments made by various healthcare professions, the Government proposes exploring to amend the Ordinance to enable PTs and OTs to provide services directly to patients under specified circumstances without a doctor's referral including (1) only patients with a condition previously diagnosed by a doctor within a pre-defined period of time; (2) a protocol-driven approach; and (3) patients under emergency or institutional settings where doctors' referral may not be practical (e.g. services at residential care homes for the elderly or people with disabilities, special schools etc.).

(A) Views on PT Direct Access in Hong Kong

1. Do you think there is a need for direct access to services of physiotherapists in Hong Kong? Why? *(Intervention characteristics)*

2. What kind of supporting evidence are there about the effectiveness of direct access to physiotherapist? *(Intervention characteristics)*

3. Do you think the PT direct access will work in Hong Kong? *(Intervention characteristics)*

(B) Design of Direct Access Model

4. What are your views on the scope and conditions for allowing direct access to services of physiotherapists in Hong Kong? Any safeguard measures? *(Intervention characteristics)*

   Probe:
   - *Settings*
   - *Clients*
   - *Patients can produce proof of diagnosis from a registered medical practitioner within the last 12 months*
   - *Compliance with clinical protocol or cross-disciplinary collaboration arrangement promulgated by authorised bodies*
   - *Provide emergencies and other circumstances endorsed by the SMP Council*
   - *Insurance*

5. How well do you think the proposed direct access model can be adapted in Hong Kong to meet local needs? *(Intervention characteristics)*
   - Any features in the proposed direct access model that will facilitate adoption/ implementation? *(Intervention characteristics)*
   - What kind of changes need to be made to the design of the proposed direct access model to facilitate implementation/ adaptation? i.e. parameters/ components for direct access? *(Intervention characteristics)*

(C) Implementation Process

6. What kind of changes and support are necessary/ anticipated to facilitate adaptation at your institution or at the system level? *(Inner setting)*

   Probe:
   Your institution
   - *Training/ Program syllabus*
   - *Clinical governance*
   - *Availability of guidelines for referral*
   - *Service monitoring*

   At system level
   - *Changes to PT Board and SMPC*
   - *Legislative amendments*
   - *Code of Practice*
   - *Incentives to engage different stakeholders*
   - *Public education / Information and materials about direct access to services* of physiotherapists are made available to different stakeholders

7. How do you think the health seeking behaviour/ culture will affect the implementation of the PT direct access? *(Inner setting)*

8. How well/ ready do you think other stakeholders (i.e. public, PTs, other healthcare professionals, employers) will adapt to PT direct access? Any barriers anticipated? *(Characteristics of individual)*

   Probe:
   - Public needs and knowledge, health seeking behaviour
   - Skills and training of PTs
   - Clarity over the role
   - Inter-professional collaborations

9. How complicated is it to execute PT direct access in Hong Kong? Any facilitators or barriers anticipated? *(Intervention characteristics)*

(D) Impact of Direct Access to PT Services

10. What is the extent of impact of PT Direct Access Model (Intervention goals)? *(Implementation Outcome)*

    Probe:
    - Patient safety & quality of service
    - Availability/ accessibility
    - Efficiency / timeliness
    - Cost effectiveness
    - Accountability & transparency
    - Manpower planning

Development of the Implementation Strategies for the Policy of Direct Access (Patient Self-Referral) to Physiotherapists in Primary Care -

Discussion Guide (Taking reference to Consolidated Framework for Implementation Research)

For Representatives from Patient Advocacy Groups

Background

Both the Chief Executive's Policy Address 2021 and 2022 (Para 135 and Para 82 respectively) specified the need to allow the public to access direct services provided by physiotherapists without a doctor's referral, as part of the initiatives to strengthen primary healthcare.

Based on the issues raised in the Policy Address, in October 2021, the Working Group on Implementation of Modified Referral System for PT Services (WG) under the Physiotherapists Board (PT Board) formulated a proposal on "Direct Access Model for Physiotherapy Service in Hong Kong", and submitted to the Supplementary Medical Professions Council (SMP) Council for discussion and endorsement in February 2023. The SMP Council wrote to the HHB in February 2023, summarising its members' views on the direct access arrangement for PTs while enclosing the proposal that the working group under the PT Board submitted to the SMP Council. Taking into account the suggestions of the SMP Council and the earlier comments made by various healthcare professions, the Government proposes exploring to amend the Ordinance to enable PTs and OTs to provide services directly to patients under specified circumstances without a doctor's referral including (1) only patients with a condition previously diagnosed by a doctor within a pre-defined period of time; (2) a protocol-driven approach; and (3) patients under emergency or institutional settings where doctors' referral may not be practical (e.g. services at residential care homes for the elderly or people with disabilities, special schools etc.).

(A) Views on PT Direct Access in Hong Kong

1. Do you think there is a need for direct access to services of physiotherapists in Hong Kong? Why? *(Intervention characteristics)*

2. What kind of supporting evidence are there about the effectiveness of direct access to physiotherapist? *(Intervention characteristics)*

3. Do you think the PT direct access will work in Hong Kong? *(Intervention characteristics)*

(B) Design of Direct Access Model

4. What are your views on the scope and conditions for allowing direct access to services of physiotherapists in Hong Kong? *(Intervention characteristics)*

Probe:
- *Settings*
- *Competency of a physiotherapist conducting direct access*
- *Scope of practice and circumstances:*
  referring the patient to a MP is necessary, i.e.
    a. *patient exhibits signs of symptoms beyond the scope of practice of a PT, after 30 calendar days or 10 visits from the initiation of a physiotherapy plan of intervention;*
    b. *patient seeks physiotherapy services for the same or similar condition within 6 months of being discharged by the physiotherapist*

  Exemptions to the time restriction on duration of physiotherapy intervention, i.e.
    a. *Cases for health promotion, wellness, fitness, and maintenance purposes.*
    b. *Cases for workplace ergonomics.*
    c. *Cases for prevention of injuries, impairments, disabilities, and functional limitations.*
    d. *Residents in residential care facilities.*
    e. *Students with chronic disabilities or special needs in school-based settings when the service is being provided for problems or symptoms associated with the chronic disabilities or special needs.*
    f. *Patients previously diagnosed with a chronic neuromuscular or developmental condition when the service is being provided for problems or symptoms associated with that previously diagnosed condition.*

5. How well do you think the proposed direct access model by the Working Group can be adapted in Hong Kong to meet local needs? *(Intervention characteristics)*
   - Any features in the proposed direct access model that will facilitate adoption/ implementation? *(Intervention characteristics)*
   - What kind of changes need to be made to the design of the proposed direct access model to facilitate implementation/ adaptation? i.e. parameters/ components for direct access? *(Intervention characteristics)*

(C) Implementation Process

6. What kind of changes and support are necessary/ anticipated to facilitate adaptation? *(Inner setting)*

   Probe:
   - *Changes to PT Board and SMPC*
   - *Legislative amendments*
   - *Code of Practice*
   - *Clinical governance*
   - *Availability of guideline for referral*

- *Incentives to engage different stakeholders*
- *Public education / Information and materials about direct access to services of physiotherapists are made available to different stakeholders*

7. How do you think the health seeking behaviour/ culture will affect the implementation of the PT direct access? *(Inner setting)* Specifically any kind of incentives and support are necessary to engage patients to self-refer to physiotherapy services? *(Inner setting)*

8. To what extent were the needs and preferences of the public considered when designing the direct access model? *(Outer setting)*

9. How well/ ready do you think other stakeholders (i.e. PTs, other healthcare professionals, employers) will adapt to PT direct access? Any barriers anticipated? *(Characteristics of individual)*

   Probe:
   - Skills and training of PTs
   - Clarity over the role
   - Inter-professional collaborations

10. How complicated is it to execute direct access to services of physiotherapists in Hong Kong? Any facilitators or barriers anticipated? *(Intervention characteristics)*

(D) Impact of Direct Access to PT Services

11. What is the extent of impact of PT Direct Access Model (Intervention goals)? *(Implementation Outcome)*

    Probe:
    - Patient safety & quality of service
    - Availability/ accessibility
    - Efficiency / timeliness
    - Cost effectiveness
    - Accountability & transparency
    - Manpower planning
