# Supplementary material for: Barriers and facilitators of direct access model to physiotherapy: intervention design and implementation strategies using the consolidated framework for implementation research
Source: Arch Public Health. 2026 Mar 12;84:80. doi: 10.1186/s13690-026-01882-7 (PMC13094042; doi:10.1186/s13690-026-01882-7)
Supplement: Supplementary file 2 — Supplementary Material 2. [file 13690_2026_1882_MOESM2_ESM.pdf]

Development of the Implementation Strategies for the Policy of Direct Access (Patient Self-Referral) to Physiotherapists in Primary Care -

Discussion Guide (Taking reference to Consolidated Framework for Implementation Research)

For Adopters (Patients ever consulted physiotherapists)

Background

Both the Chief Executive's Policy Address 2021 and 2022 (Para 135 and Para 82 respectively) specified the need to allow the public to access direct services provided by physiotherapists without a doctor's referral, as part of the initiatives to strengthen primary healthcare.

Based on the issues raised in the Policy Address, in October 2021, the Working Group on Implementation of Modified Referral System for PT Services (WG) under the Physiotherapists Board (PT Board) formulated a proposal on "Direct Access Model for Physiotherapy Service in Hong Kong", and submitted to the Supplementary Medical Professions Council (SMP) Council for discussion and endorsement in February 2023. The SMP Council wrote to the HHB in February 2023, summarising its members' views on the direct access arrangement for PTs while enclosing the proposal that the working group under the PT Board submitted to the SMP Council. Taking into account the suggestions of the SMP Council and the earlier comments made by various healthcare professions, the Government proposes exploring to amend the Ordinance to enable PTs and OTs to provide services directly to patients under specified circumstances without a doctor's referral including (1) only patients with a condition previously diagnosed by a doctor within a pre-defined period of time; (2) a protocol-driven approach; and (3) patients under emergency or institutional settings where doctors' referral may not be practical (e.g. services at residential care homes for the elderly or people with disabilities, special schools etc.).

(A) Views on PT Direct Access in Hong Kong

1. What are your experiences with using physiotherapist services?

   Probe:
   - *Reasons for using the service e.g. acute or chronic pain*
   - *Referral experience*
   - *Patient autonomy*
   - *Choice of service provider*
   - *Quality of service*
   - *Availability/ accessibility, efficiency, accountability, transparency etc.*

2. Do you think there is a need for direct access to services of physiotherapists in Hong Kong? Why? *(Intervention characteristics)*

(B) Design and Implementation Process of Direct Access Model

3. If PT direct access is allowed, what circumstances/ which conditions will you consider to go to see a PT or doctor? *(Intervention characteristics)*

4. If PT direct access is allowed, what criteria will you use to choose the PT services? Will you consider the qualifications/ competency of a physiotherapist before directly approaching the PTs? *(Intervention characteristics)*

5. How well does PT direct access fit with your health-seeking practice? *(inner setting)*

6. The Government is considering the implementation of PT direct access, how much do you know about this PT direct access model or its implementation? What are your views on this proposal? Any safeguard measures are required? *(Characteristics of individual)*

   Probe:
   - *Settings*
   - *Patients can produce proof of diagnosis from a registered medical practitioner within the last 12 months*
   - *Compliance with clinical protocol or cross-disciplinary collaboration arrangement promulgated by authorised bodies*
   - *Provide emergencies and other circumstances endorsed by the SMP Council*
   - *Pre-requisite requirement/ competency of a physiotherapist conducting direct access including Mandatory continuing professional development*

7. How confident/ ready are you to adapt to this PT direct access model? Any barriers anticipated? *(Characteristics of individual)*

   Probe:
   - *Public knowledge*

- *Competency of PT (knowledge and training)*
- *Patient safety*
- *Accountability*
- *Oversight & control of professional practice (Regulation)*

8. What kind of incentives and support are necessary to engage you to self-refer to physiotherapy services? *(Inner setting)*

(C) Impact of Direct Access to PT Services

9. What is the extent of impact of PT Direct Access Model (Intervention goals)? *(Implementation Outcome)*

   Probe:
   - Patient safety & quality of service
   - Availability/ accessibility
   - Efficiency / timeliness
   - Cost effectiveness
   - Accountability & transparency
   - Manpower planning

Development of the Implementation Strategies for the Policy of Direct Access (Patient Self-Referral) to Physiotherapists in Primary Care -

Discussion Guide (Taking reference to Consolidated Framework for Implementation Research)

For Providers (Physiotherapists)

Background

Both the Chief Executive's Policy Address 2021 and 2022 (Para 135 and Para 82 respectively) specified the need to allow the public to access direct services provided by physiotherapists without a doctor's referral, as part of the initiatives to strengthen primary healthcare.

Based on the issues raised in the Policy Address, the Working Group on Implementation of Modified Referral System for PT Services (the Working Group) under the Physiotherapists Board (PT Board) started to formulate the direct access model for implementation in October 2021. A proposal on "Direct Access Model for Physiotherapy Service in Hong Kong" was compiled by the Working Group which was then submitted to the PT Board in September 2022 and the Supplementary Medical Professions Council (SMPC) on 1 February 2023 for discussion and endorsement. The SMP Council wrote to the HHB in February 2023, summarising its members' views on the direct access arrangement for PTs while enclosing the proposal that the working group under the PT Board submitted to the SMP Council. Taking into account the suggestions of the SMP Council and the earlier comments made by various healthcare professions, the Government proposes exploring to amend the Ordinance to enable PTs and OTs to provide services directly to patients under specified circumstances without a doctor's referral including

(1) Patients can produce proof of diagnosis from a registered medical practitioner within the last 12 months;
(2) Compliance with clinical protocol or cross-disciplinary collaboration arrangement promulgated by authorized bodies; and
(3) Emergencies and other circumstances endorsed by the SMP Council

(A) Views on PT Direct Access in Hong Kong

1. Could you describe how you assess a patient who has been referred by a doctor before commencing treatment? How do you treat your patient?

2. Do you think there is a need for direct access to services of physiotherapists in Hong Kong? Why? How would it help the current situation? *(Intervention characteristics)*

(B) Design of Direct Access Model

3. What are your views on the scope and conditions for allowing direct access to services of physiotherapists in Hong Kong? Any safeguard measures? *(Intervention characteristics)*

   Probe:
   - *Settings*
   - *Patients can produce proof of diagnosis from a registered medical practitioner within the last 12 months*
   - *Compliance with clinical protocol or cross-disciplinary collaboration arrangement promulgated by authorised bodies*
   - *Provide emergencies and other circumstances endorsed by the SMP Council*
   - *Pre-requisite requirement/ competency of a physiotherapist conducting direct access including Mandatory continuing professional development*
   - *Insurance*

4. How well do you think the proposed direct access model can be adapted in Hong Kong to meet local needs? *(Intervention characteristics)*
   - Any features in the proposed direct access model that will facilitate adoption/ implementation? *(Intervention characteristics)*
   - What kind of changes need to be made to the design of the proposed direct access model to facilitate implementation/ adaptation? i.e. parameters/ components for direct access? *(Intervention characteristics)*

(C) Implementation Process

5. What kind of changes and support are necessary/ anticipated to facilitate your adaptation? *(Inner setting)*

   Probe:
   - *Changes to PT Board and SMPC*
   - *Legislative amendments*
   - *Code of Practice*
   - *Clinical governance*
   - *Availability of guideline for referral*
   - *Incentives to engage different stakeholders*

- *Public education / Information and materials about direct access to services of physiotherapists are made available to different stakeholders*

6. How confident are you to implement PT direct access at your setting/ clinic? E.g. understanding of the intervention/ its implementation, knowledge/ training required? *(Characteristics of individual)*

7. To what extent do you work with other healthcare professionals especially in cases that requires multidisciplinary care or beyond your scope of practice, including doctors, radiographers etc.? How would the PT direct access model impact on the inter-professional collaborations? *(Inner setting)*

8. How do you think the health seeking behaviour/ culture will affect the implementation of the PT direct access? *(Inner setting)* How will you engage patients to self-refer to physiotherapy services? *(Process)*

9. How complicated is it to execute PT direct access in Hong Kong? Any facilitators or barriers anticipated? *(Intervention characteristics)*

(D) Impact of Direct Access to PT Services

10. What is the extent of impact of PT Direct Access Model (Intervention goals)? *(Implementation Outcome)*

    Probe:
    - Patient safety & quality of service
    - Availability/ accessibility
    - Efficiency / timeliness
    - Cost effectiveness
    - Accountability & transparency
    - Manpower planning

Development of the Implementation Strategies for the Policy of Direct Access (Patient Self-Referral) to Physiotherapists in Primary Care -

Discussion Guide (Taking reference to Consolidated Framework for Implementation Research)

For Referrers (Medical Doctors)

Background

Both the Chief Executive's Policy Address 2021 and 2022 (Para 135 and Para 82 respectively) specified the need to allow the public to access direct services provided by physiotherapists without a doctor's referral, as part of the initiatives to strengthen primary healthcare.

Based on the issues raised in the Policy Address, the Working Group on Implementation of Modified Referral System for PT Services (the Working Group) under the Physiotherapists Board (PT Board) started to formulate the direct access model for implementation in October 2021. A proposal on "Direct Access Model for Physiotherapy Service in Hong Kong" was compiled by the Working Group which was then submitted to the PT Board in September 2022 and the Supplementary Medical Professions Council (SMPC) on 1 February 2023 for discussion and endorsement. The SMP Council wrote to the HHB in February 2023, summarising its members' views on the direct access arrangement for PTs while enclosing the proposal that the working group under the PT Board submitted to the SMP Council. Taking into account the suggestions of the SMP Council and the earlier comments made by various healthcare professions, the Government proposes exploring to amend the Ordinance to enable PTs and OTs to provide services directly to patients under specified circumstances without a doctor's referral including (1) only patients with a condition previously diagnosed by a doctor within a pre-defined period of time; (2) a protocol-driven approach; and (3) patients under emergency or institutional settings where doctors' referral may not be practical (e.g. services at residential care homes for the elderly or people with disabilities, special schools etc.).

(A) Views on PT Direct Access in Hong Kong

1. What is your current practice in referring patients to private PT services?

   Probe:
   Are PTs available in your practice?
   Do you have a fixed PT working for you, may be at another site?

2. Do you think there is a need for direct access to PT services in Hong Kong? Why? *(Intervention characteristics)*

(B) Design of Direct Access Model

3. What are your views on the scope and conditions for allowing direct access to services of physiotherapists in Hong Kong? Any safeguard measures? *(Intervention characteristics)*

   Probe:
   - *Settings*
   - *Patients can produce proof of diagnosis from a registered medical practitioner within the last 12 months*
   - *Compliance with clinical protocol or cross-disciplinary collaboration arrangement promulgated by authorised bodies*
   - *Provide emergencies and other circumstances endorsed by the SMP Council*
   - *Pre-requisite requirement/ competency of a physiotherapist conducting direct access including Mandatory continuing professional development*
   - *Insurance*

4. How well do you think the proposed direct access model can be adapted in Hong Kong to meet local needs? *(Intervention characteristics)*
   - Any features in the proposed direct access model that will facilitate adoption/ implementation? *(Intervention characteristics)*
   - What kind of changes need to be made to the design of the proposed direct access model to facilitate implementation/ adaptation? i.e. parameters/ components for direct access? *(Intervention characteristics)*

(C) Implementation Process

5. What kind of changes and support are necessary/ anticipated to facilitate your adaptation to PT direct access? *(Inner setting)*

   Probe:
   - *Changes to PT Board and SMPC*
   - *Legislative amendments*
   - *Code of Practice*

- *Clinical governance*
- *Availability of guideline for referral*
- *Incentives to engage different stakeholders*
- *Public education / Information and materials about direct access to services of physiotherapists are made available to different stakeholders*
- *?establish referral channels from PT back to a medical practitioner*

6. To what extent do you work with other healthcare professionals especially in cases that requires multidisciplinary care? How would the PT direct access model impact on the inter-professional collaborations? *(Inner setting)*

7. How do you think the health seeking behaviour/ culture will affect the implementation of the PT direct access? *(Inner setting)*

8. How complicated is it to execute PT direct access in Hong Kong? Any facilitators or barriers anticipated? *(Intervention characteristics)*

(D) Impact of Direct Access to PT Services

9. What is the extent of impact of PT Direct Access Model (Intervention goals)? *(Implementation Outcome)*

   Probe:
   - Patient safety & quality of service
   - Availability/ accessibility
   - Efficiency / timeliness
   - Cost effectiveness
   - Accountability & transparency
   - Manpower planning, e.g. need to employ more PTs in my clinic
