# Supplementary material for: Barriers and facilitators of direct access model to physiotherapy: intervention design and implementation strategies using the consolidated framework for implementation research
Source: Arch Public Health. 2026 Mar 12;84:80. doi: 10.1186/s13690-026-01882-7 (PMC13094042; doi:10.1186/s13690-026-01882-7)
Supplement: Supplementary file 3 — Supplementary Material 3. [file 13690_2026_1882_MOESM3_ESM.pdf]

Supplementary File: COREQ reporting standards checklist

**No. Item**	**Guide questions/description**	**Key informant interviews Reported on Page # Line #**	**Focus group discussions Reported on Page # Line #**
**Domain 1: Research team and reflexivity**			
Personal Characteristics			
1. Interviewer/facilitator	Which author/s conducted the interview or focus group?	Page 8 Line 5-6	Page 9 Line 16-17
2. Credentials	What were the researcher's credentials? E.g. PhD, MD	Page 1 Line 5-7	Page 1 Line 5-7
3. Occupation	What was their occupation at the time of the study?	Page 1 Line 10-13, showing the resarchers' oorganization having the required information	Page 1 Line 10-13, showing the resarchers' oorganization having the required information
4. Gender	Was the researcher male or female?		
5. Experience and training	What experience or training did the researcher have?		
Relationship with participants			
6. Relationship established	Was a relationship established prior to study commencement?	Page 7 Line 16-22, showing no relationship established since they are recruited based on their expertise and experience	Page 9 Line 3-9, showing no relationshop established prior to study commencement since the participants are recruited through respective professional associations and patients groups, and univerity's mass mail system

No. Item	Guide questions/description	Key informant interviews Reported on Page # Line #	Focus group discussions Reported on Page # Line #
7. Participant knowledge of the interviewer	What did the participants know about the researcher? e.g. personal goals, reasons for doing the research	Page 8 Line 5-6	Page 9 Line 5-6, 17-18
8. Interviewer characteristics	What characteristics were reported about the interviewer/facilitator? e.g. Bias, assumptions, reasons and interests in the research topic	Page 8 Line 5-6	Page 9 Line 5-6, 17-18
**Domain 2: study design**			
Theoretical framework			
9. Methodological orientation and Theory	What methodological orientation was stated to underpin the study? e.g. grounded theory, discourse analysis, ethnography, phenomenology, content analysis	Page 6 Line 18-25, Page 7 Line 1-5, & Page 9 Line 24, & Page 10 Line 1	Page 6 Line 18-25, Page 7 Line 1-5, & Page 9 Line 24, & Page 10 Line 1
Participant selection			
10. Sampling	How were participants selected? e.g. purposive, convenience, consecutive, snowball	Page 7 Line 22-24	Page 8 Line 24-25, & Page 9 Line 1-3
11. Method of approach	How were participants approached? e.g. face-to-face, telephone, mail, email	Page 7 Line 24	Page 9 Line 3-5
12. Sample size	How many participants were in the study?	Page 11 Line 2-7	Page 11 Line 12-16
13. Non-participation	How many people refused to participate or dropped out? Reasons?	No	No
Setting			

**No. Item**	**Guide questions/description**	**Key informant interviews Reported on Page # Line #**	**Focus group discussions Reported on Page # Line #**
14. Setting of data collection	Where was the data collected? e.g. home, clinic, workplace	Page 8 Line 4-5	Page 9 Line 11-12
15. Presence of non-participants	Was anyone else present besides the participants and researchers?	No	No
16. Description of sample	What are the important characteristics of the sample? e.g. demographic data, date	Page 11 Line 1-7	Page 11 Line 14-24, Page 12 1-6, & Table 1
Data collection			
17. Interview guide	Were questions, prompts, guides provided by the authors? Was it pilot tested?	Page 8 Line 9-11	Page 9 Line 12-16
18. Repeat interviews	Were repeat interviews carried out? If yes, how many?	No	No
19. Audio/visual recording	Did the research use audio or visual recording to collect the data?	Page 8 Line 11-12	Page 9 Line 19-20
20. Field notes	Were field notes made during and/or after the interview or focus group?	Page 8 Line 6-7	Page 9 Line 17-18
21. Duration	What was the duration of the interviews or focus group?	Page 8 Line 4	Page 9 Line 11
22. Data saturation	Was data saturation discussed?	Page 8 Line 12-13	Page 9 Line 19
23. Transcripts returned	Were transcripts returned to participants for comment and/or correction?	No	No
**Domain 3: analysis and findings**			
Data analysis			

No. Item	Guide questions/description	Key informant interviews Reported on Page # Line #	Focus group discussions Reported on Page # Line #
24. Number of data coders	How many data coders coded the data?	Page 10 Line 3-4	Page 10 Line 3-4
25. Description of the coding tree	Did authors provide a description of the coding tree?	Page 10 Line 2-15	Page 10 Line 2-15
26. Derivation of themes	Were themes identified in advance or derived from the data?	Page 10 Line 2-15	Page 10 Line 2-15
27. Software	What software, if applicable, was used to manage the data?	Page 10 Line 1-2	Page 10 Line 1-2
28. Participant checking	Did participants provide feedback on the findings?	No	No
Reporting			
29. Quotations presented	Were participant quotations presented to illustrate the themes/findings? Was each quotation identified? e.g. participant number	Table 3	Table 3
30. Data and findings consistent	Was there consistency between the data presented and the findings?	Page 10 Line 2-15	Page 10 Line 2-15
31. Clarity of major themes	Were major themes clearly presented in the findings?	Page 12 Line 10-16, & Table 2	Page 12 Line 10-16, & Table 2
32. Clarity of minor themes	Is there a description of diverse cases or discussion of minor themes?	Page 12 Line 10-16, & Table 2	Page 12 Line 10-16, & Table 2
